# An efficient floral dipping transformation method for the metal hyperaccumulator *Noccaea caerulescens*

**DOI:** 10.1007/s11248-026-00506-8

**Published:** 2026-07-14

**Authors:** Jitpanu Yamjabok, Elisabeth C. M. van der Heijden, Maarten Koornneef, Henk Schat, Mark G. M. Aarts

**Affiliations:** 1 Laboratory of Genetics, Wageningen University and Research, Droevendaalsesteeg 1, 6708 PB Wageningen, The Netherlands; 2 Present Address: National Center for Genetic Engineering and Biotechnology, 113 Thailand Science Park, Phahonyothin Road, Khlong Luang District, Pathum Thani, 12120 Thailand; 3 Present Address: Systems Biology Lab, AIMMS, Vrije Universiteit, De Boelelaan 1108, 1081 HZ Amsterdam, The Netherlands

**Keywords:** *Noccaea caerulescens*, Metal hyperaccumulator, *Agrobacterium tumefaciens*, Transformation, Early flowering, *FLOWERING LOCUS C (FLC)*

## Abstract

*Noccaea caerulescens* is a metal hyperaccumulator plant species with the capacity to accumulate high concentrations of zinc (Zn), cadmium (Cd) and nickel (Ni). Several candidate genes have so far been associated with accumulation and tolerance of these metals, but gene function analysis has been cumbersome in the absence of an efficient stable plant transformation method for *N. caerulescens*. A previously identified mutation in the *FLOWERING LOCUS C* gene, conferring early flowering to the calamine accession St. Felix de Pallières, has been introgressed in the genetic background of five different genotypes, originating from the calamine populations Clough Wood (CLW) and Le Blémard (BLE), the ultramafic population Cira (CIR), and the non-metallicolous populations St. Baudille (SBD) and Werschmatt (WER). *Agrobacterium tumefaciens* mediated floral dipping transformation was employed in five introgression lines in these backgrounds and in *Arabidopsis thaliana* as control. Three introgression lines, of genetic backgrounds CLW, BLE and SBD, were successfully transformed, achieving an average transformation efficiency ≥ 0.29% for each background. A reproducible, stable transformation method based on floral dipping is presented here as an improvement on previously reported methods. The early flowering *N. caerulescens flc* mutation can be readily introgressed into different genetic backgrounds to facilitate genetic transformation studies, which is expected to provide a highly desired extra genetic tool to study gene function analysis in the metal hyperaccumulator *N. caerulescens*.

## Introduction

*Noccaea caerulescens* (J. & C. Presl) F.K. Mey., previously named *Thlaspi caerulescens*, is an annual, temperate plant species, that can grow and survive on soil rich in cadmium (Cd), lead (Pb) and zinc (Zn) (calamine soil) or in nickel (Ni) (ultramafic or serpentine soil), due to its very high metal tolerance (Assunção et al. 2003; Baker et al. 1994; Mohtadi et al. 2012; Richau and Schat 2009). In addition, this species can accumulate elevated amounts of Cd, Zn, Pb and Ni in its aerial parts, which is known as metal hyperaccumulation (Brooks et al., 1998). This has raised an interest to study *N. caerulescens* and explore the biological mechanisms and genetic architectures underlying metal hyperaccumulation and tolerance (Assunção et al. 2003; Milner and Kochian 2008). An understanding of these traits is expected to contribute successfully to several useful applications, such as for phytoremediation, using plants to clean metal-contaminated soil, especially for arable land (Ashraf et al. 2010; Zhao and McGrath 2009); for phytomining, using plants to extract valuable metals from anthropogenically metal-polluted or naturally metal-rich lands (Brooks et al. 1998; Chaney and Baklanov 2017) or for biofortification, improving the nutritional value of food to combat human micronutrient deficiency (Zhao and McGrath 2009). *N. caerulescens* is an attractive model metal hyperaccumulator species as it is a diploid plant (2n = 14) that is fully self-compatible and easily outcrossed, which makes breeding and genetic research relatively easy (Assunção et al. 2003). Belonging to the Brassicaceae family, with several well investigated species, notably the general plant model *Arabidopsis thaliana* with ~ 88% sequence identify in coding regions (Rigola et al. 2006) and with transcriptome sequences available for *N. caerulescens* (Halimaa et al. 2014; Y-F. Lin 2014), there is also ample opportunity for gene identification.

*N. caerulescens* has been investigated for physiological, biochemical and genetic aspects of metal accumulation and tolerance, resulting in the identification of several genes and quantitative trait loci (QTL) involved with accumulation and tolerance of Zn, Cd and Ni (Assunção et al. 2001, 2006; Deniau et al. 2006; Halimaa et al. 2014; Mortel et al. 2008; Rigola et al. 2006; van de Mortel et al. 2006). However, these candidate genes and QTLs are rarely explored due to scarcity of reverse genetic tools in *N. caerulescens*.

Genetic transformation is a widely used reverse genetic tool in several species. Three transformation methods have been reported for *N. caerulescens*. The first one based on *Agrobacterium tumefaciens* mediated floral dipping (Peer et al. 2003), one on *A. tumefaciens*-mediated transformation upon co-cultivation of explants in tissue culture (Guan et al. 2008); and one based on hairy root transformation using *Agrobacterium rhizogenes* (Lin et al. 2016). Each method experiences different challenges. Floral dipping, initially developed and optimised for *A. thaliana* (Clough and Bent 1998; Feldmann and David Marks 1987) requires flowering plants, which is a lengthy procedure in *N. caerulescens* since it requires 2–3 months of vernalisation to flower, after a 2–3 month pre-flowering growth period. Transformation by tissue culture is more complicated than the publication suggests, or requiring considerable skills, as this method has only been reported once. Although also only reported once for *N. caerulescens*, transformation by *A. rhizogenes* is more commonly used for other species but has the disadvantage that only chimeric transgenic plants can be produced, with a transgenic root system, and a non-transgenic shoot. Transgenic plants are therefore limited to one genetic generation, and difficult to maintain for a long time. Of the three methods, the floral dipping transformation method seems to be the most attractive to develop for further use in future *N. caerulescens* studies, as, other than requiring flowering plants, it does not appear to require considerable technical skills, and will result in stable transgenic plants, which can be propagated by self-pollination. Especially if the generation of flowering plants bearing many inflorescences suitable for dip-inoculation could be optimized, this could become a valuable tool for reverse genetic studies in *N. caerulescens*.

*N. caerulescens* is a winter bi-annual species, with a life cycle around 6–9 months. It first involves a vegetative phase of 2–3 months, a vernalization phase of 2–3 months, growing at a low temperature to induce flowering, and a pollination and seed-ripening phase of 2–3 months, to produce mature seeds that can be stored for longer time. To generate stable, homozygous T_2_ generation plants, will therefore need 12–18 months, and a climate-controlled growth room that can be set at ~ 5 °C, for vernalization. Experiments have been performed in the past, to generate mutants that do not require vernalisation to flower, a trait also referred to as ‘early flowering.’ Using fast neutron mutagenesis, Lochlainn et al. (2011) generated an early flowering mutant in the Ganges accession, and Wang et al. (2020) generated several early flowering mutants using an EMS-mutagenesis approach. The accession ‘St. Felix de Pallières’ (SF) has been recommended as a promising genotype for studying metal hyperaccumulation (Peer et al. 2003, 2006). Among the different early flowering mutants identified by Wang et al. (2020), three were identified to contain a mutation in the *FLOWERING LOCUS C* (*FLC*) gene, and one in the *SHORT VEGETATIVE PHASE* (*SVP*) gene.

The *flc-1* mutant contains a G to A point mutation in the third exon of *FLC*, that results in a splice variant and rearrangement of the third exon in the coding sequence (Wang et al. 2020). The *flc-1* mutant shows pronounced early flowering, at 51 days after sowing, without vernalization (Wang et al. 2020) and high fecundity when grown under well-fertilized conditions. Recently, a collection of 86 accessions of *N. caerulescens* has been described, which provides a wealth of genetic and phenotypic variation for metal related and morphological traits (van der Zee et al. 2021). These can be used to generate an interesting genetic resource to improve fecundity and self-fertility of *flc-1* via conventional intercrossing.

This study presents the generation of fertile, early flowering *N. caerulescens* inter-accession recombinants, and an optimized, simple protocol for the generation of transgenic plants from such recombinants through floral dipping.

## Methodology

### Plant material and generation of early flowering F_2_/F_3_ plants

Twenty-three *N. caerulescens* accessions, known for their fecundity and self-fertility, were used to cross with the early flowering *flc-1* mutant, originally identified in an EMS-mutagenized population in the St. Felix de Pallières (SF) background. All accessions originate from different populations in Europe and are inbred for at least five generations (Table S1). Seed propagation of all genotypes has been performed in a frost-free (≥ 5 °C) greenhouse at the Wageningen University campus (51°59′46.4 ′′N 5°39′29.4 ′′E), during October to July in the past years.

The early flowering *flc-1* mutant was crossed as father to each accession. Before crossing, the seeds of the accessions were surface-sterilised and pre-germinated in modified half-strength Hoagland’s solution: 3 mM KNO_3_, 2 mM Ca(NO_3_)_2_.4H_2_O, 1 mM NH_4_H_2_PO_4_, 0.5 mM MgSO_4_.7H_2_O, 1 µM KCl, 25 µM H_3_BO_3_, 2 µM MnSO_4_.4H_2_O, 2 µM ZnSO_4_.7H_2_O, 0.1 µM CuSO_4_.5H_2_O, 0.1 µM (NH_4_)6Mo_7_O_24_.4H_2_O and 20 µM Fe-EDDHA (*N,N′*-ethylenediamine-di(O-hydroxyphenylacetic acid), and was buffered at pH = 5.5 with 2 mM MES (2-[*N*-morpholino]-ethanesulfonic acid) solution. After stratification at 4 °C for four days, the seeds were transferred to a climate-controlled growth room, set at 20 °C, 15 h/9 h light/dark, and 70% humidity. After radicle emergence, the seeds were sown, and seedlings were grown in pots with a mix of fertilised peat and sand (Table S2) in a frost-free (> 5 °C) greenhouse from October onwards. Vernalized plants started to flower in March/April the next year, by which the crosses could be conducted. The F_1_ seeds of each cross were harvested in July and sown again in October, to flower after vernalization. Flowers were self-pollinated to generate F_2_ seeds following the same procedure as before.

To identify the early flowering plants among the segregating F_2_ progeny of each cross, seeds were pre-germinated as described above and then sown on 4 × 4 × 4 cm rockwool blocks (Grodan, The Netherlands), weekly supplied with nutrient solution (Table S3). These plants were grown in a climate-controlled growth room set at 21 °C, 15 h/9 h light/dark, and 70% humidity. The flowering F_2_ (~ 25% if the crosses were successful), displaying high fecundity, high fertility and segregation of morphological characteristics (e.g. rosette diameter and plant height) were selected for production of F_3_ seeds by self-pollination.

### Transformation by floral dipping

F_3_ seeds from early flowering F_2_ plants of the crosses with 25% flowering plants were pre-germinated as described above. These plants were grown in pots with a mix of fertilised peat and sand in a climate-controlled growth cabinet (Weiss Technik Nederland B.V., The Netherland) at 20 °C, 15 h/9 h light/dark and 70% humidity. Three plants per F_3_ progeny were used for this experiment.

A Silwet L-77 sensitivity test was performed on *N. caerulescens* inflorescences. For this, three concentrations of Silwet L-77 (Lehle seeds, USA): 0.02% v/v, 0.05% v/v and 0.1% v/v, were applied on the tested inflorescence twice, with a 7-day interval, to mimic a double-dipping transformation. The highest concentration that did not cause necrotic tissues on leaves and inflorescences was chosen for floral dipping transformation.

*A. tumefaciens* strain GV3101 carrying a modified plasmid was used for this experiment. This plasmid carried the *OLEOSIN1-*Red Fluorescent Protein (*OLE1-RFP*) marker construct, shortly called pFAST-R02 (Shimada et al. 2010), enabling fluorescence in transformed seeds, as well as an enhanced Green Fluorescent Protein (eGFP) marker construct, as shown in Figure S1. An infiltration medium was prepared as described by Clough and Bent (1998) with 5% sucrose and Silwet L-77 (Lehle seeds, USA) at the concentration identified in the sensitivity test. For floral dipping, the inoculation medium was added to a 50-mL conical tube and the inflorescences with long peduncles were submerged in the medium. For the inflorescences with short peduncles, which remained very close to the soil surface, the inoculation medium was pipetted directly on those inflorescences. Then, the dipped plants were placed in closed plastic bags to maintain humidity and covered with a black plastic bag overnight. On the next day, the bag was removed from the dipped plants. These plants were dipped with the same procedure for the second time a week later to increase the transformation efficiency (Davis et al. 2009). The plants were grown for a further 10 weeks until siliques were ripe and dry. The ripe seeds were harvested from siliques and stored in plastic bags. *A. thaliana* introgression line IL-A4 was used as a positive control for transformation. IL-A4 carries a small Col-0 introgression in a Chromosome Substitution Line (CSL) 32 genetic background, but largely resembles Ler (Wijnen 2019).

### Screening for transformed seeds and stable transformation

To identify transformed seeds, T_1_ seeds was screened for an RFP signal using a fluorescence stereo microscope Axio Zoom.V16 (Carl Zeiss, Germany) equipped with a dsRED filter. The images were captured in Zeiss Zen 2.1 software (Carl Zeiss, Germany). The transformation efficiency is calculated as:

1 $${\mathrm{Transformation}}\,{\mathrm{efficiency}} = { }\frac{{{\mathrm{number}}\,{\mathrm{of}}\,{\mathrm{fluorescent}}\,{\mathrm{seeds}}}}{{{\mathrm{total}}\,{\mathrm{number}}\,{\mathrm{of}}\,{\mathrm{seeds}}}}{ } \times 100$$

Transformed T_1_ seeds were grown on 0.3% modified half-strength Hoagland’s agar plate. After three weeks, germinated seedlings were observed for GFP signal using the same fluorescence stereo microscope, equipped with a GFP filter.

## Result

### Early flowering traits in fertile *N. caerulescens* backgrounds

**Fig. 1:**
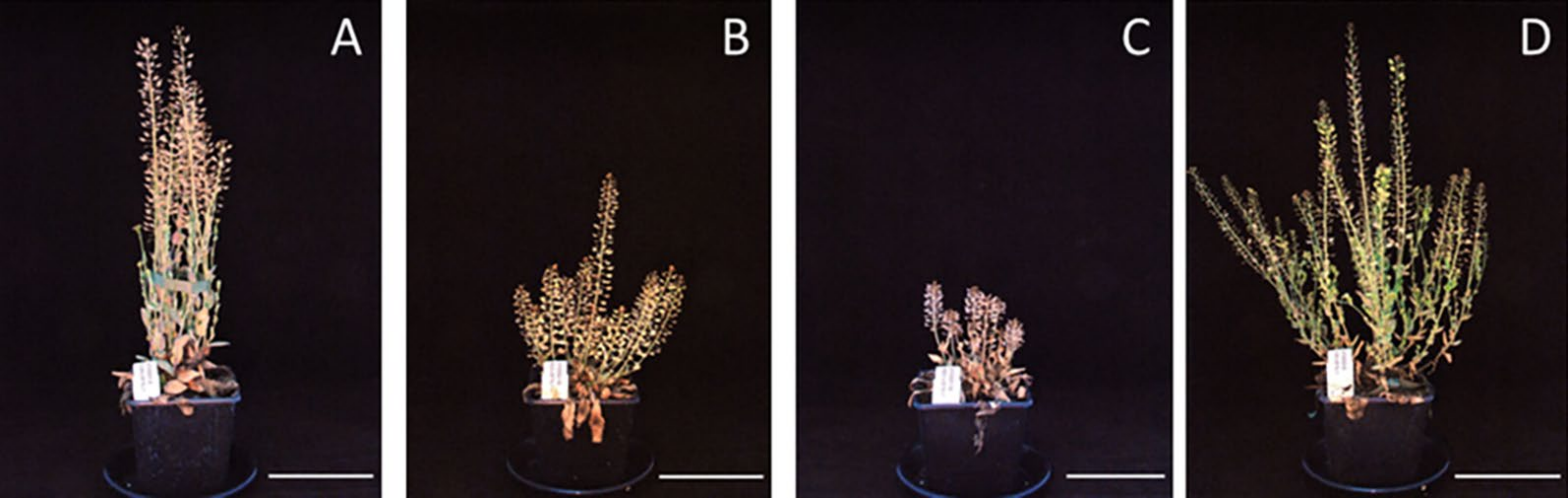
*N. caerulescens *intercrossed populations were made between the flc-1 mutant and four maternal backgrounds: Clough Wood (CLW) (**A**), Le Blémard (BLE) (**B**), Saint Baudille (SBD) (**C**) and Cira (CIR) (**D**). Scale bar 10 cm

The SF *flc-1* mutant is not very self-fertile, due to the low efficiency in self-pollination (Wang et al. 2020). This drawback makes SF *flc-1* not a very attractive genotype to use to develop flower-dip transformation in *N. caerulescens*. Fortunately, there are several *N. caerulescens* accessions that are highly self-fertile, autonomously producing many inflorescences and high seed yield. Therefore, to develop fertile *N. caerulescens* backgrounds with the early flowering trait, crosses were made between early flowering mutant *flc-1* (Wang et al. 2020) and 23 self-fertile *N. caerulescens* accessions. F_1_ plants were grown and vernalized to generate F_2_ seeds. Of these, only five F_2_ lines, with accessions Clough Wood (CLW), Le Blémard (BLE), Cira (CIR), Saint Baudille (SBD) (Fig. 1) and Werschmatt (WER) as paternal parent, showed the early flowering trait to segregate, with early flowering plants producing abundant seeds. A few others segregated early flowering plants but failed to produce seeds. The flowering time of the F_2_ plants with high fecundity ranged from 44.5 to 58.5 days after sowing (DAS). The SBD maternal genotype conferred the earliest average flowering time at 44.5 DAS (Table 1). The F_3_ progeny seeds of these five lines were obtained upon natural self-pollination of individual plants, providing enough seeds for further propagation and transformation.

**Table 1:** Flowering characteristics of fertile F_2_ progenies upon crossing with the SF *flc-1* mutant

Maternal background	Acronym	Ecotype^a^	% Flowering plants	Ratio non-flowering: flowering plants	Average flowering time (days after sowing)^b^	Autonomous seed setting
La Calamine	LC	CAL	53.34	7:8	58.5 ± 3.5	No
Lellingen	LE	NM	0	15:0	0	
Monte Prinzera	MP	ULT	0	15:0	0	
Krušné Hory Mountains	KHM	NM	80	3:12	55 ± 4.06	No
Moravsko-Slezské Beskydy Mountains	MSB	NM	26.67	11:4	48.75 ± 6.76	No
Durfort	DUR	CAL	0	15:0	0	
Clough Wood	CLW	CAL	26.67	11:4	49.75 ± 5.76	Yes
Puerto de Aralla	ARA	NM	0		0	
Le Blémard	BLE	CAL	26.67	11:4	54 ± 6.48	Yes
Cira	CIR	ULT	26.67	11:4	55.75 ± 4.15	Yes
Copenhagen	COP	NM	0		0	
Kuopio	KUO	NM	6.67	14:1	57 ± 0	No
Höör	HOO	NM	26.67	11:4	55 ± 3.27	No
Werschmatt	WER	NM	60	6:9	53.33 ± 1.25	Yes
Horná Roveň	HRO	CAL	66.67	5:10	58 ± 2.94	No
Valle Gargassino	VGG	ULT	6.67	14:1	52 ± 0	No
Auxelles	AUX	CAL	13.33	13:2	55 ± 3	No
Fresse sur Moselle	FSM	NM	60	6:9	54.75 ± 2.68	No
Col du Marchairuz	MAR	NM	20	12:3	52.5 ± 3.5	No
Tête de Ran	TDR	NM	0	15:0		
Rozenburg	ROZ	Calamine	0	15:0		
Saint Baudille	SBD	NM	26.67	11:4	44.5 ± 1.5	Yes
Špania Dolina	SDO	CAL	0	15:0		

^a^NM: Non-metallicolous; CAL = Calamine; ULT = Ultramafic

^b^Data are presented as mean ± standard deviation of three replications

For the transformation experiment, F_3_ plants were grown in 7-L pots with a mixture of fertilised peat and sand, to give abundant inflorescences and flowers. Flowering time ranged between 48 and 64 DAS and these plants took approximately 20 weeks after sowing to yield mature ripe seeds. The CLW maternal background was the earliest flowering at 48.3 DAS and yielded the highest number of harvested seeds (Table 2). The CIR maternal background had long fragile stems, perhaps because it was most sensitive to the relatively low irradiance, and a high rate of seed abortion (Fig. 1D).

**Table 2:** Flowering time and seed yield of selected F_3_ populations

Maternal background	Average flowering time (days after sowing)	Average seed number
CLW	48.3 ± 0.5	545 ± 266.7
BLE	64.3 ± 0.5	361.7 ± 19.3
CIR	64 ± 0.8	188.3 ± 101.7
SBD	55.2 ± 3.3	121.7 ± 15.4
WER	< 48	250 ± 71.6

Data are presented as mean ± standard deviation of three replications

### *N. caerulescens* inflorescence sensitivity to Silwet L-77

**Fig. 2:**
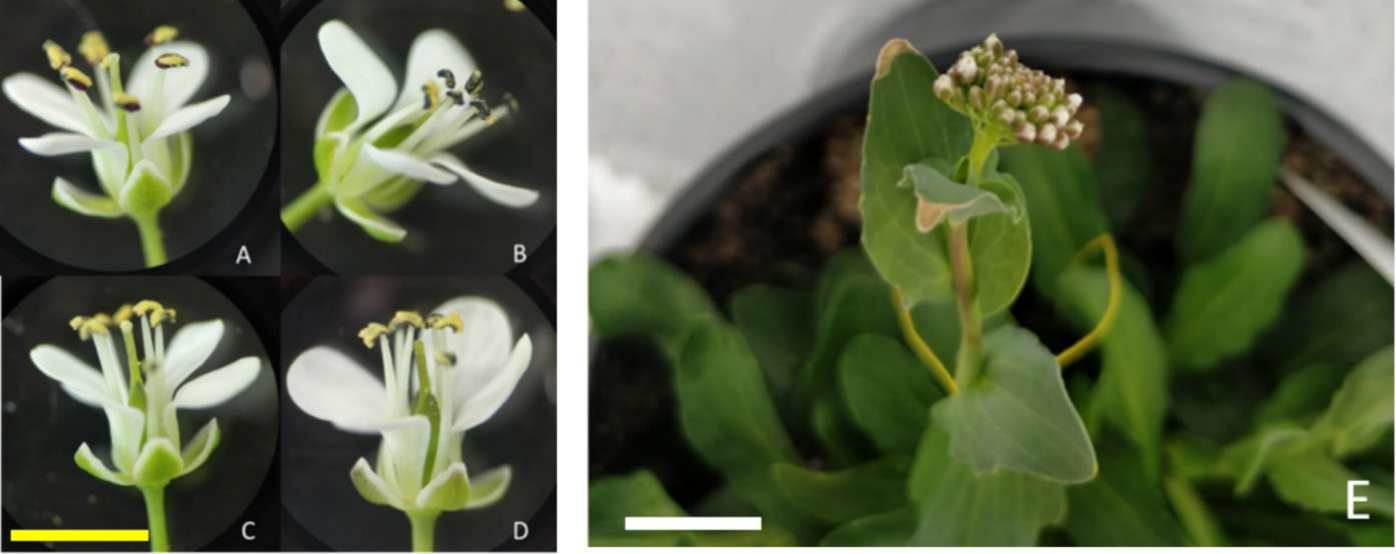
*N. caerulescens* flowers did not show any necrotic response to Silwet L-77 below 0.1% v/v, but bracts do. **A**, 0 v/v %, **B**, 0.05 v/v%, **C**, 0.02 v/v % and **D**, 0.1 v/v% Silwet L-77 applied on inflorescences. **E**: The bracts of N. caerulescens treated with 0.05 v/v % Silwet L-77 show necrotic edges. This image was taken seven days after the second Silwet L-77 application. Yellow and white scale bars indicate 1 mm and 1 cm, respectively

The concentration of Silwet L-77 in the infiltration medium has impact on the transformation efficiency of *A. thaliana* upon floral dipping (Clough and Bent 1998). A pilot experiment was therefore performed to evaluate the sensitivity of *N. caerulescens* inflorescences to Silwet L-77. The inflorescences did not show any sign of sensitivity (e.g. necrotic tissue) to any of these Silwet L-77 concentrations. However, on the leaves, necrosis was found at 0.05% v/v and 0.1%v/v Silwet L-77 (Fig. 2). Based on these results, we concluded that 0.02% v/v Silwet L-77 in the infiltration medium would be the best concentration to try for *N. caerulescens* transformation.

### Successful floral dipping transformation in three maternal backgrounds

Floral dipping transformation of *N. caerulescens* was attempted using *A. tumefaciens* strain GV3101 carrying the pFAST-R02 eGFP plasmid. This modified plasmid contains two efficient visible marker genes, suitable for non-destructive marker gene expression and selection: a) *pOLE1-RFP* (the original selectable marker of plasmid pFAST-R02) and b) *p35S-GFP*. The *pOLE1-RFP* allows screening for successful transformation in the seed stage. In addition, the *p35S-GFP* serves as an additional marker for transformation, with GFP expression expected to be visible at most stages of plant development and growth.

**Fig. 3:**
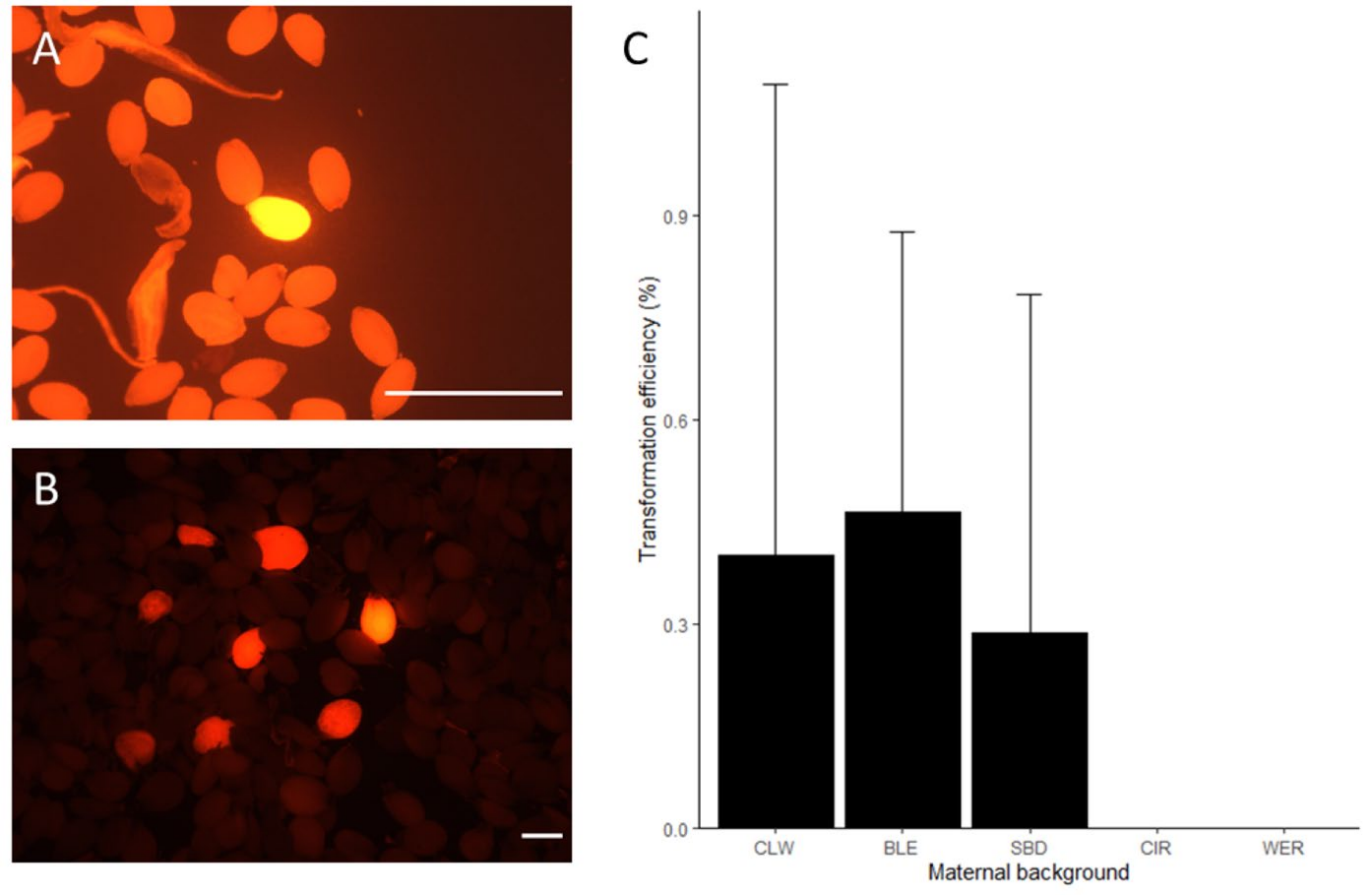
Successfully transformed seeds of *A. thaliana *(**A**) and *N. caerulescens *of the Clough Wood (CLW) maternal background (**B**), exhibiting Red Fluorescent Protein-positive fluorescence. Scale bar is 1 mm. (**C**) Transformation efficiencies for different maternal backgrounds (CLW, BLE, SBO, CIR and WER; Suppl. Table S1). The bars represent standard deviation (n = 3)

*A. thaliana* was used as positive control for floral dip transformation. *A. thaliana* T_1_ seeds exhibited RFP expression (Fig. 3A), and the average transformation efficiency was established at 0.35%. For *N. caerulescens* flower dip transformation, the infiltration medium was applied using two approaches: a) by submerging inflorescences with a long peduncle into the infiltration medium; or b) by pipetting the infiltration medium directly on the inflorescence in case plants had a short peduncle (such as for BLE, which is semi-dwarf). The T_1_ progeny of maternal backgrounds CLW, BLE and SBD provided seeds with red fluorescence, as evidence for stable transformation (Fig. 3B). Progeny of plants with WER and CIR maternal backgrounds did not give any red fluorescent seeds. The average transformation efficiencies were 0.40% (CLW), 0.46% (BLE) and 0.29% (SBD) (Fig. 3C). The GFP signal was also observed in T_1_ seeds but only in the RFP-positive seeds with a BLE maternal background. This indicated that GFP is not very efficient for selection in seeds for floral dip transformation, most likely as the 35S promoter was not very effective to drive expression of GFP in *N. caerulescens* seeds.

**Fig. 4:**
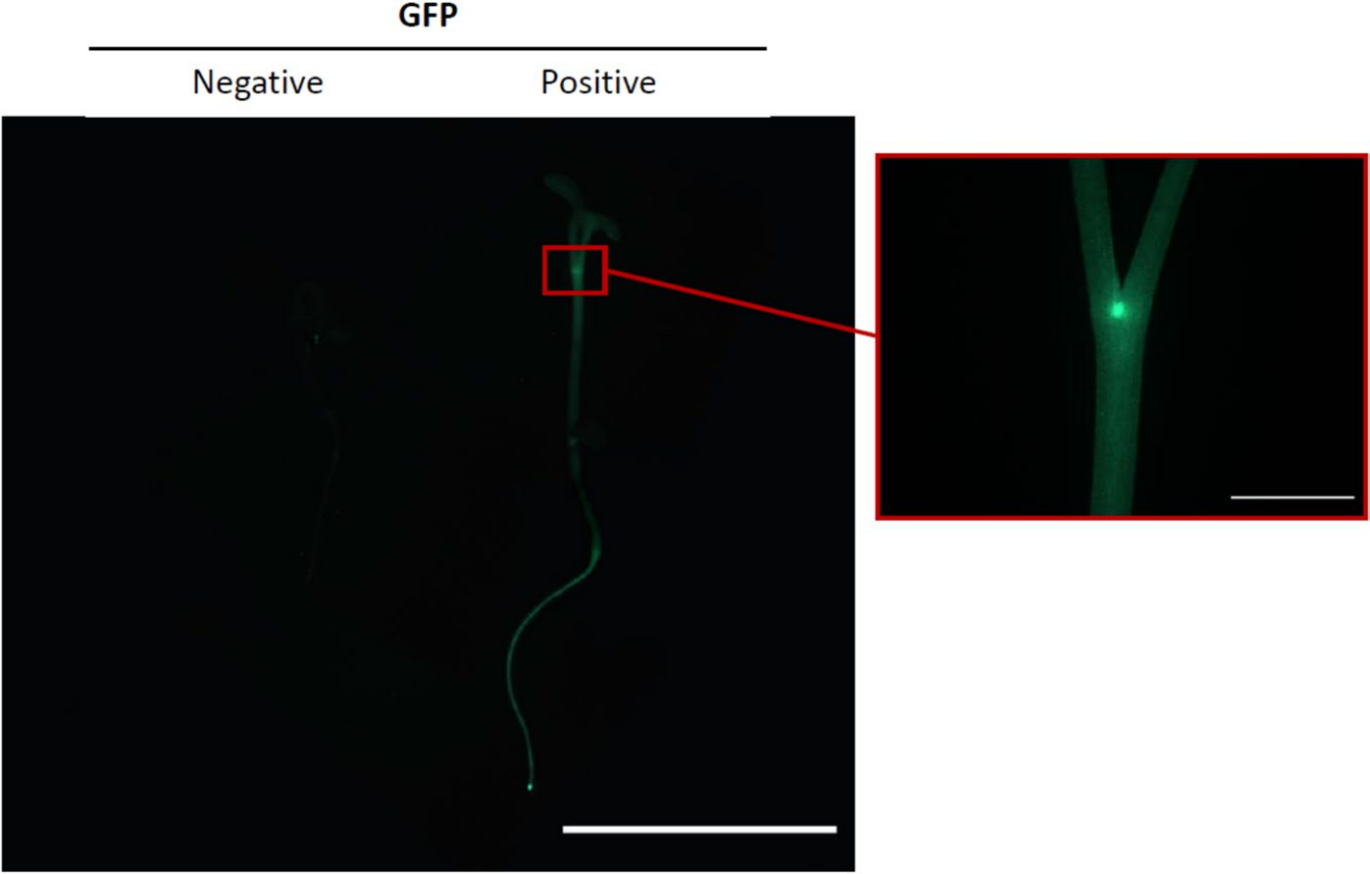
An example of a GFP-negative and a GFP-positive seedling in the T_1_ of CLW maternal background (left; scale bar is 10 cm). The meristematic region exhibiting a strong GFP-positive signal (right; scale bar 1 cm)

To further validate the stable expression of inserted genes integrated in the host genome upon transformation, the expression of p35S-GFP was determined in the germinated seedlings. Indeed, all T_1_ seedlings originating from seeds selected for RFP expression, also showed green fluorescence. The intensity of green fluorescence was strongest in the meristematic regions (Fig. 4; Supplementary Figure S2). Overall, these results show that introduction of the *flc-1* early flowering mutation in different highly self-fertile genetic backgrounds, yielded three early flowering lineages suitable for flower-dip transformation of *N. caerulescens* with efficiencies up to 0.46%.

## Discussion

*N. caerulescens* has been proposed as a model species for studying metal hyperaccumulation and hypertolerance for two decades (Assunção et al. 2003). However, so far, reverse genetic studies on this species were very difficult to perform, in the absence of a simple transformation method giving stable transformants that can be genetically propagated through seeds. While stable genetic transformation of *N. caerulescens* has been reported twice (Guan et al. 2008; Peer et al. 2003), somehow these methods were not considered to be attractive or successful, as no follow-up use has been reported until now. It was not clear if this was due to the difficulties in accomplishing the right regeneration and selection conditions in tissue culture, or the very lengthy procedure needed for flower dip transformation, requiring a 2–3 months vernalization period for plants to induce flowering, or if there were further complications, perhaps caused by differences in transformation ability between different *N. caerulescens* genotypes. The recent isolation of well-characterized early flowering mutants, such as the *flc* and *svp* mutants, identified upon EMS-mutagenesis in the SF background, offered the opportunity to significantly reduce the floral-dipping transformation procedure, abolishing the need for vernalization for plants to flower. Unfortunately, the SF genetic background is not optimal for this approach. While plants in the field show no sign of reduced fecundity, in a controlled growth environment, such as a climate room or greenhouse, plants display low fecundity (Wang et al. 2020, 2022). This is not due to genetic defects, but more likely associated with the flower architecture shared by many accessions in the Cevennes region in France where this accession originates from (Mousset et al. 2016). Due to this flower architecture, anthers and stigma remain at a distance upon opening of flower buds, favouring cross-pollination by insects. Manual pollination could be used to overcome this, but it would make the transformation procedure undesirably labour intensive. We therefore set out to introduce the early flowering *flc-1* mutation in different, highly self-fertile, *N. caerulescens* accessions. Considering the poor self-fertility of SF, we decided to use the *flc-1* SF genetic background as father in the crosses, which in retrospect was not the best choice, as we could not make use of the recessive *flc* phenotype to select against unintended self-pollinations. Out of 23 crosses made, only five segregated early flowering plants that produced sufficient seeds. This low number of successful crosses probably relates to problems in emasculating the flowers of the mother plants. Considering the long time to induce flowering in wild-type accessions, we did not attempt to make the reciprocal crosses, but such could be done in the future, to evaluate additional backgrounds. There is no obvious restriction to introgress *flc-1* into other genetic backgrounds, but then it will be advisable to use *flc-1* SF as a mother, rather than a father in the crosses. This will reveal already in the F1 if the crosses have been successful (i.e. late flowering, vs. early flowering if the cross failed), and not just in the F_2_, as we experienced.

Of these, four showed the expected recessive segregation of approximately 25% early flowering plants in the F_2_, with one (WER) giving an excess of early flowering plants. We grew the plants on rock wool, rather than potting mix or soil, but this was not the best choice of substrate. Several plants grew poorly, and the stressful conditions may even have induced early flowering, irrespective of *flc*. We noticed in the past that for some of the accessions, poor conditions may induce the onset of flowering, but in that case the plants are poor and flowers are often not fertile.

Our study demonstrates that introducing the early flowering trait indeed facilitates successful and efficient genetic transformation of *N. caerulescens*. In the previous study reporting successful transformation of *N. caerulescens* by floral dipping, herbicide tolerance (conferred by the *bar* gene) was used as selectable marker along with GFP as visible expression marker for stable transformation. Overall transformation efficiencies then reached approximately 0.6% (Peer et al. 2006), which is slightly higher than the transformation efficiencies we found, ranging from 0.29 to 0.46%. Among the accessions tried for floral dip transformation by Peer et al. (2003) were also SF and BLE genotypes, likely to originate from the same, large, populations present at the sites. Interestingly, transformation by spray inoculation resulted in abortion of all flowers for BLE (Peer et al. 2003), something we did not observe at all for the BLE *flc-1* F_3_ plants, with a transformation efficiency of 0.46%. Rather than spray-inoculation, we applied the inoculum by pipetting, which may explain for the differences in success. Peer et al. (2003) also used 0.02% Silwet, as we did. Of course there may also be an effect of genetic background. We used introgressions of *flc-1* in the BLE background, and could, by chance, have selected for plants carrying alleles favouring floral dipping, as there is likely to still be genetic variation within the population. We selected for F_2_ plants of each cross that were early flowering (a SF trait) and providing high fecundity (a non-SF trait). Although we did not further determine plant fertility, crossing SF with high-fecundity *N. caerulescens* backgrounds certainly yielded ample high-fecundity *flc-1* progeny, suggesting that the poor fertility observed for SF is a recessive rather than a dominant trait.

The vernalization requirement to induce flowering and the difficulties in stable transformations have been serious limitations in developing *N. caerulescens* as a convenient model for metal hyperaccumulation and tolerance (Assunção et al. 2003; Lochlainn et al. 2011; Wang et al. 2020), certainly compared to *A. thaliana*, which can be reproduced up to four generations in the 24–36 weeks it may take for wild-type *N. caerulescens* to complete one seed-to-seed generation. The F_2_ and F_3_ progeny of the inter-accession crosses we made, took ~ 20 weeks to complete one seed-to-seed generation. This is longer than reported for the original *flc-1* mutant (~ 16 weeks to complete its life cycle), except for the CLW maternal background, but considerably faster than their wild-type backgrounds. *FLC* is one of the key proteins regulating induction to flowering in Brassicaceae species (Michaels and Amasino 1999). The increase in flowering time of *flc-1* progeny upon introgression of the mutant allele in two other genetic backgrounds, suggests that there is additional variation for flowering time that affects the *flc-1* phenotype, although the identity of the genes underlying this variation is not yet known. The generated *flc-1* F2 and F3 plants can complete their life cycle without any further special requirements such as special temperatures or light regimes. This provides a versatile and cost-effective system facilitating transgenic studies in *N. caerulescens*. Establishing stable transformed F_2_ lines can be achieved within one year.

Genetic transformation in plants may be influenced by genetic background, with genotype specificity to be known to play a crucial role to determine the success of a transformation experiment (Ghedira et al. 2013). Our results identified the CLW x SF *flc-1* progeny as the most promising resource for *N. caerulescens* transformation, with high fecundity, fast early flowering and long peduncle. CLW originates from calamine soil and exhibits Zn and Ni accumulation characteristics comparable to those of SF (van der Zee et al., 2021). The Cd accumulation properties of CLW are not known yet, but SF is a good Cd accumulator (Koshevnikova et al., 2020), which could provide good Cd accumulation in the different F2/F3 progenies. SBD, the other background we could transform by floral dipping, originates from non-metallicolous soil. It is known to have average Zn and Cd tolerance (among other *N. caerulescens* accessions), and high Ni tolerance, as well as average Zn and Ni accumulation and high Cd accumulation, exceeding that of SF or BLE (Kozhevnikova et al. 2020). BLE is known to show high Zn and Cd tolerance, with average Ni tolerance, and relatively low Zn and Cd accumulation, with average Ni accumulation. Unfortunately, we were unsuccessful to transform the CIR and WER backgrounds, which is particularly unfortunate as both are collected at or close to ultramafic outcrops, respectively in Galicia, Spain, and the Vosges, France. The CIR background turned out to be hard to transform as the peduncles of the plants were more rigid than those of others, and prone to break upon covering upon floral dipping, leading to progressive loss of inflorescences, and transformed seeds, upon handling. Furthermore, the CIR *flc-1* F_3_ plants yielded a low average number of seeds and may be more affected by the Silwet than other plants. However, further refinements in the method may prove to be successful in establishing also this background as suitable for floral-dip transformation. For WER, we are not sure why the transformation was unsuccessful. Seed yield and peduncle length or rigidity is not different from the successfully transformed plants. It may therefore be that somehow WER is less susceptible for *A. tumefaciens* infection than the others, which is also known for some *A. thaliana* accessions (Clough and Bent 1998; Ghedira et al. 2013). Since we did not evaluate a large number of plants, increasing this may well show that also CIR and WER *flc-1* plants can be transformed.

From the work on *A. thaliana*, there are several factors known to contribute to the success of *Agrobacterium*-meditated floral dip transformation, which can be further optimised. We used the common *A. tumefaciens* strain GV3101 for transformation, which was more efficient than other strains, such as LBA4404, C58C1, LMG201 or LMG62, in *A. thaliana* Col-0 (Ghedira et al. 2013). We did not investigate other strains, but some of those may be more effective in *N. caerulescens*.

## Conclusion

This study reports a substantial improvement of one of the few reports on successful transformation of the metal hyperaccumulator *N. caerulescens*, by abolishing the vernalization requirement to flower upon introgression of the early-flowering-conferring *flc-1* mutation in the genetic background of three accessions, CLW, BLE and SBD. This transformation system will be a useful resource for future reverse genetic studies on metal accumulation and tolerance.

## Data Availability

The datasets used and/or analysed during the current study are available from the corresponding author on reasonable request.
